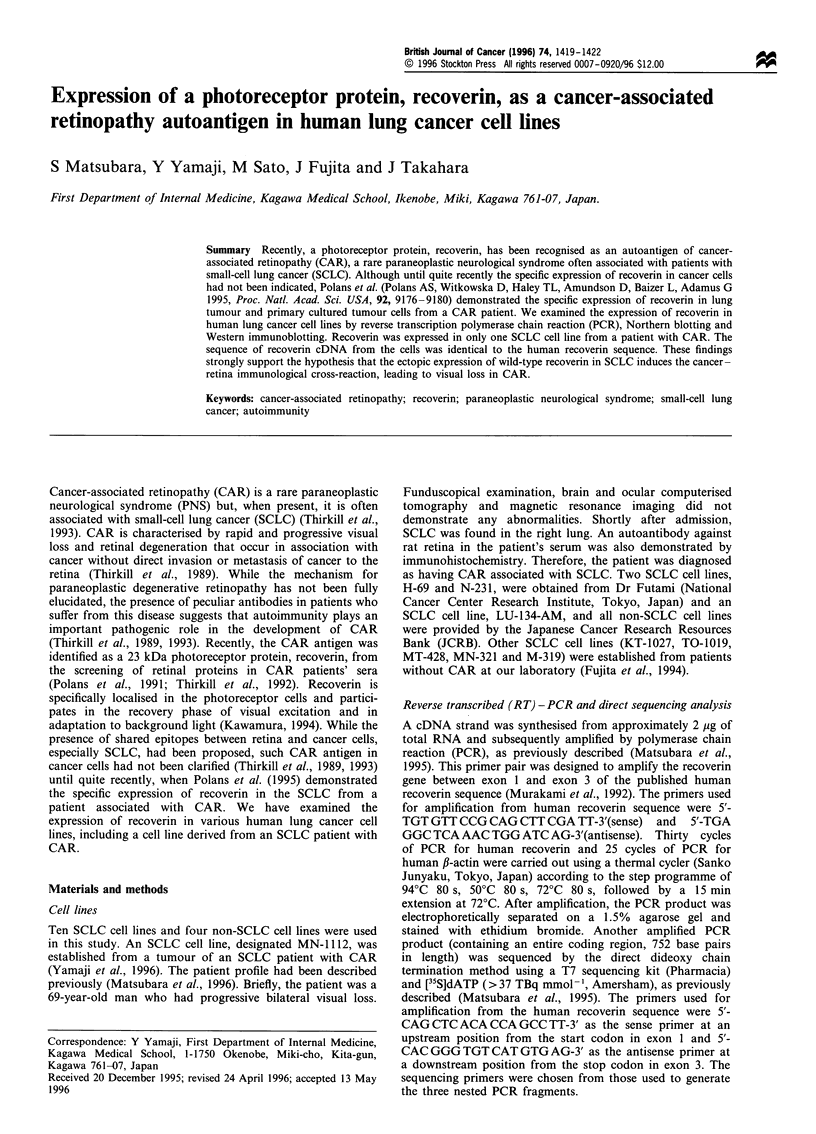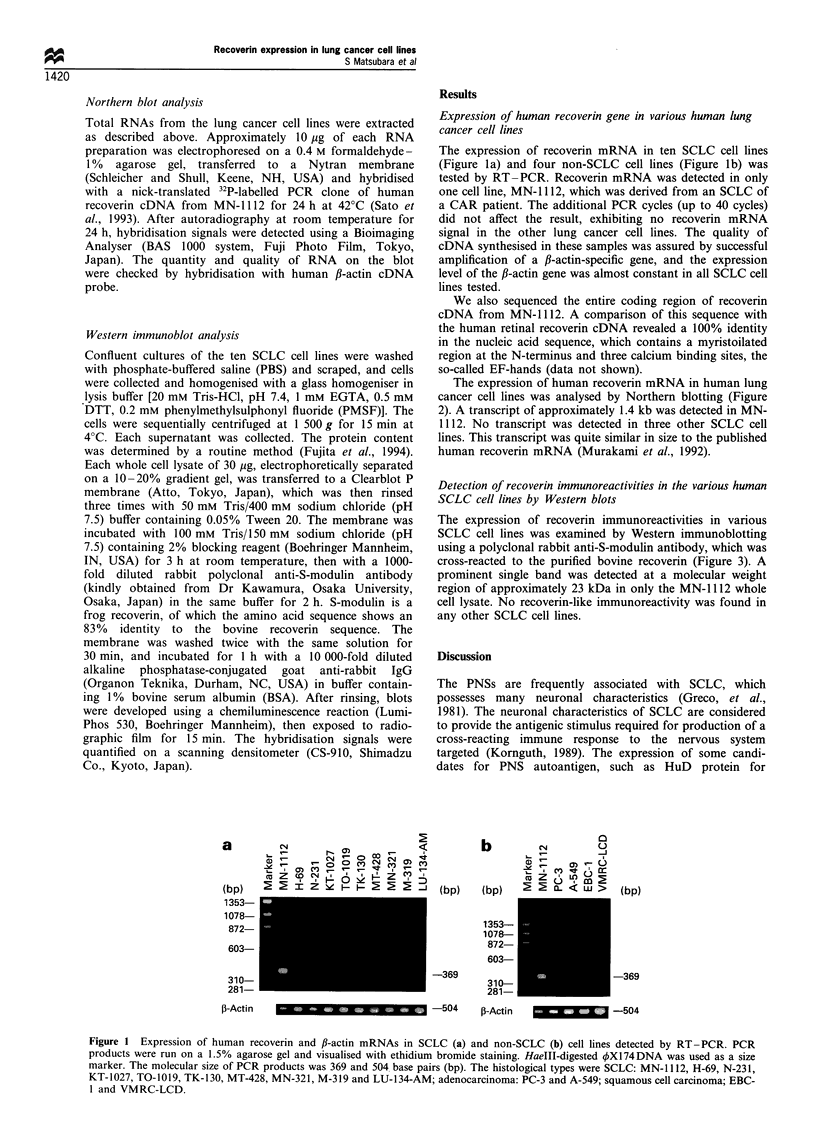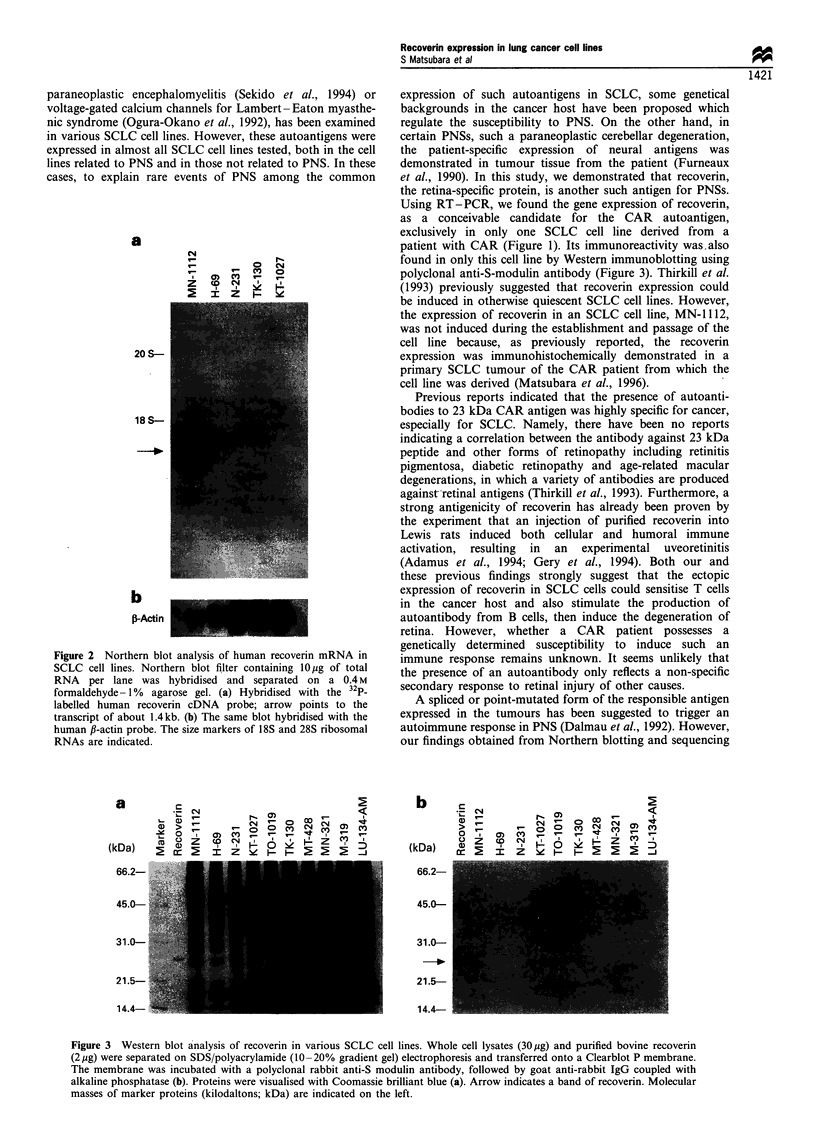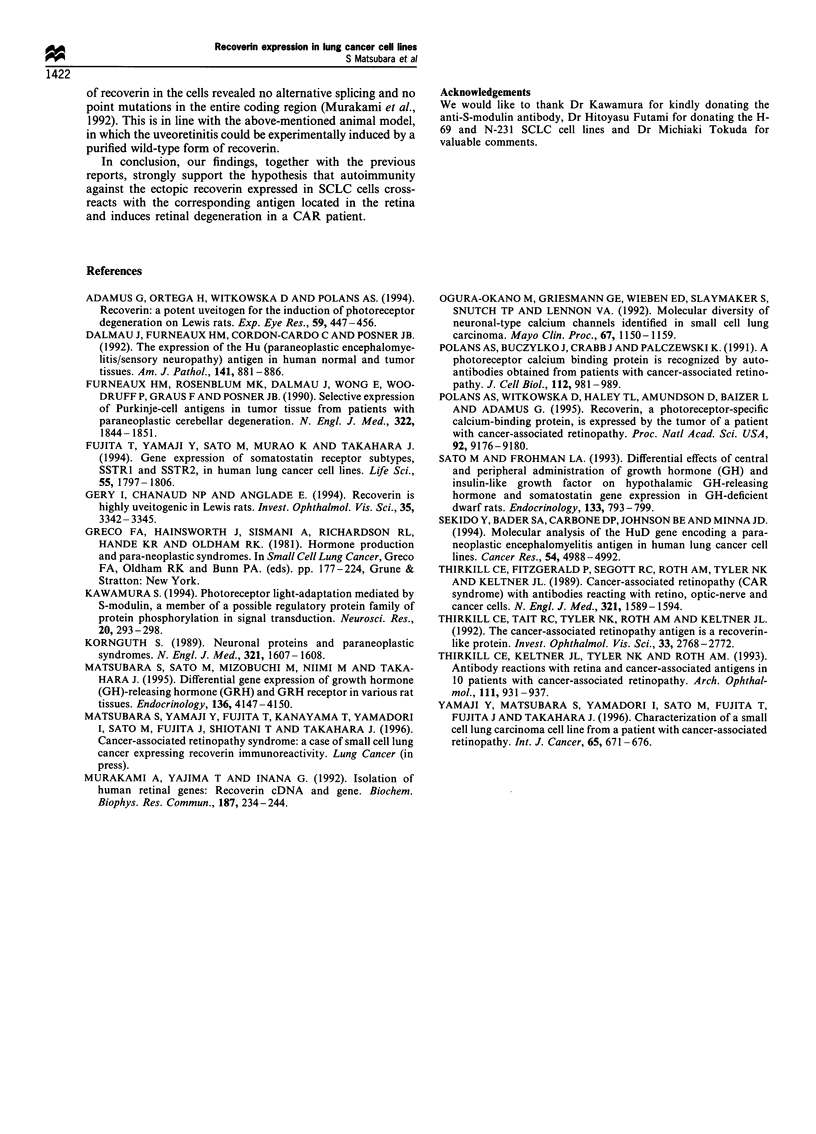# Expression of a photoreceptor protein, recoverin, as a cancer-associated retinopathy autoantigen in human lung cancer cell lines.

**DOI:** 10.1038/bjc.1996.558

**Published:** 1996-11

**Authors:** S. Matsubara, Y. Yamaji, M. Sato, J. Fujita, J. Takahara

**Affiliations:** First Department of Internal Medicine, Kagawa Medical School, Japan.

## Abstract

**Images:**


British Journal of Cancer (1996) 74, 1419-1422
© 1996 Stockton Press All rights reserved 0007-0920/96 $12.00

### Expression of a photoreceptor protein, recoverin, as a cancer-associated retinopathy autoantigen in human lung cancer cell lines

S Matsubara, Y Yamaji, M Sato, J Fujita and J Takahara

*First Department of Internal Medicine, Kagawa Medical School, Ikenobe, Miki, Kagawa 761-07, Japan.*

**Summary** Recently, a photoreceptor protein, recoverin, has been recognised as an autoantigen of cancer-associated retinopathy (CAR), a rare paraneoplastic neurological syndrome often associated with patients with small-cell lung cancer (SCLC). Although until quite recently the specific expression of recoverin in cancer cells had not been indicated, Polans *et al.* (Polans AS, Witkowska D, Haley TL, Amundson D, Baizer L, Adamus G 1995, *Proc. Natl. Acad. Sci. USA*, **92,** 9176-9180) demonstrated the specific expression of recoverin in lung tumour and primary cultured tumour cells from a CAR patient. We examined the expression of recoverin in human lung cancer cell lines by reverse transcription polymerase chain reaction (PCR), Northern blotting and Western immunoblotting. Recoverin was expressed in only one SCLC cell line from a patient with CAR. The sequence of recoverin cDNA from the cells was identical to the human recoverin sequence. These findings strongly support the hypothesis that the ectopic expression of wild-type recoverin in SCLC induces the cancer–retina immunological cross-reaction, leading to visual loss in CAR.

**Keywords:** cancer-associated retinopathy; recoverin; paraneoplastic neurological syndrome; small-cell lung cancer; autoimmunity

Cancer-associated retinopathy (CAR) is a rare paraneoplastic neurological syndrome (PNS) but, when present, it is often associated with small-cell lung cancer (SCLC) (Thirkill *et al.*, 1993). CAR is characterised by rapid and progressive visual loss and retinal degeneration that occur in association with cancer without direct invasion or metastasis of cancer to the retina (Thirkill *et al.*, 1989). While the mechanism for paraneoplastic degenerative retinopathy has not been fully elucidated, the presence of peculiar antibodies in patients who suffer from this disease suggests that autoimmunity plays an important pathogenic role in the development of CAR (Thirkill *et al.*, 1989, 1993). Recently, the CAR antigen was identified as a 23 kDa photoreceptor protein, recoverin, from the screening of retinal proteins in CAR patients' sera (Polans *et al.*, 1991; Thirkill *et al.*, 1992). Recoverin is specifically localised in the photoreceptor cells and participates in the recovery phase of visual excitation and in adaptation to background light (Kawamura, 1994). While the presence of shared epitopes between retina and cancer cells, especially SCLC, had been proposed, such CAR antigen in cancer cells had not been clarified (Thirkill *et al.*, 1989, 1993) until quite recently, when Polans *et al.* (1995) demonstrated the specific expression of recoverin in the SCLC from a patient associated with CAR. We have examined the expression of recoverin in various human lung cancer cell lines, including a cell line derived from an SCLC patient with CAR.

### Materials and methods

### *Cell lines*

Ten SCLC cell lines and four non-SCLC cell lines were used in this study. An SCLC cell line, designated MN-1112, was established from a tumour of an SCLC patient with CAR (Yamaji *et al.*, 1996). The patient profile had been described previously (Matsubara *et al.*, 1996). Briefly, the patient was a 69-year-old man who had progressive bilateral visual loss. Funduscopical examination, brain and ocular computerised tomography and magnetic resonance imaging did not demonstrate any abnormalities. Shortly after admission, SCLC was found in the right lung. An autoantibody against rat retina in the patient's serum was also demonstrated by immunohistochemistry. Therefore, the patient was diagnosed as having CAR associated with SCLC. Two SCLC cell lines, H-69 and N-231, were obtained from Dr Futami (National Cancer Center Research Institute, Tokyo, Japan) and an SCLC cell line, LU-134-AM, and all non-SCLC cell lines were provided by the Japanese Cancer Research Resources Bank (JCRB). Other SCLC cell lines (KT-1027, TO-1019, MT-428, MN-321 and M-319) were established from patients without CAR at our laboratory (Fujita *et al.*, 1994).

### *Reverse transcribed (RT) – PCR and direct sequencing analysis*

A cDNA strand was synthesised from approximately 2 $\mu g$ of total RNA and subsequently amplified by polymerase chain reaction (PCR), as previously described (Matsubara *et al.*, 1995). This primer pair was designed to amplify the recoverin gene between exon 1 and exon 3 of the published human recoverin sequence (Murakami *et al.*, 1992). The primers used for amplification from human recoverin sequence were 5'-TGT GTT CCG CAG CTT CGA TT-3'(sense) and 5'-TGA GGC TCA AAC TGG ATC AG-3'(antisense). Thirty cycles of PCR for human recoverin and 25 cycles of PCR for human $\beta$-actin were carried out using a thermal cycler (Sanko Junyaku, Tokyo, Japan) according to the step programme of 94°C 80 s, 50°C 80 s, 72°C 80 s, followed by a 15 min extension at 72°C. After amplification, the PCR product was electrophoretically separated on a 1.5% agarose gel and stained with ethidium bromide. Another amplified PCR product (containing an entire coding region, 752 base pairs in length) was sequenced by the direct dideoxy chain termination method using a T7 sequencing kit (Pharmacia) and [$^{35}$S]dATP (>37 TBq mmol$^{-1}$, Amersham), as previously described (Matsubara *et al.*, 1995). The primers used for amplification from the human recoverin sequence were 5'-CAG CTC ACA CCA GCC TT-3' as the sense primer at an upstream position from the start codon in exon 1 and 5'-CAC GGG TGT CAT GTG AG-3' as the antisense primer at a downstream position from the stop codon in exon 3. The sequencing primers were chosen from those used to generate the three nested PCR fragments.

Correspondence: Y Yamaji, First Department of Internal Medicine, Kagawa Medical School, 1-1750 Okenobe, Miki-cho, Kita-gun, Kagawa 761–07, Japan
Received 20 December 1995; revised 24 April 1996; accepted 13 May 1996

*Northern blot analysis*

Total RNAs from the lung cancer cell lines were extracted as described above. Approximately 10 $\mu g$ of each RNA preparation was electrophoresed on a 0.4 M formaldehyde–1% agarose gel, transferred to a Nytran membrane (Schleicher and Shull, Keene, NH, USA) and hybridised with a nick-translated $^{32}P$-labelled PCR clone of human recoverin cDNA from MN-1112 for 24 h at 42°C (Sato *et al*., 1993). After autoradiography at room temperature for 24 h, hybridisation signals were detected using a Bioimaging Analyser (BAS 1000 system, Fuji Photo Film, Tokyo, Japan). The quantity and quality of RNA on the blot were checked by hybridisation with human $\beta$-actin cDNA probe.

*Western immunoblot analysis*

Confluent cultures of the ten SCLC cell lines were washed with phosphate-buffered saline (PBS) and scraped, and cells were collected and homogenised with a glass homogeniser in lysis buffer [20 mM Tris-HCl, pH 7.4, 1 mM EGTA, 0.5 mM DTT, 0.2 mM phenylmethylsulphonyl fluoride (PMSF)]. The cells were sequentially centrifuged at 1 500 ***g*** for 15 min at 4°C. Each supernatant was collected. The protein content was determined by a routine method (Fujita *et al*., 1994). Each whole cell lysate of 30 $\mu g$, electrophoretically separated on a 10–20% gradient gel, was transferred to a Clearblot P membrane (Atto, Tokyo, Japan), which was then rinsed three times with 50 mM Tris/400 mM sodium chloride (pH 7.5) buffer containing 0.05% Tween 20. The membrane was incubated with 100 mM Tris/150 mM sodium chloride (pH 7.5) containing 2% blocking reagent (Boehringer Mannheim, IN, USA) for 3 h at room temperature, then with a 1000-fold diluted rabbit polyclonal anti-S-modulin antibody (kindly obtained from Dr Kawamura, Osaka University, Osaka, Japan) in the same buffer for 2 h. S-modulin is a frog recoverin, of which the amino acid sequence shows an 83% identity to the bovine recoverin sequence. The membrane was washed twice with the same solution for 30 min, and incubated for 1 h with a 10 000-fold diluted alkaline phosphatase-conjugated goat anti-rabbit IgG (Organon Teknika, Durham, NC, USA) in buffer containing 1% bovine serum albumin (BSA). After rinsing, blots were developed using a chemiluminescence reaction (Lumi-Phos 530, Boehringer Mannheim), then exposed to radiographic film for 15 min. The hybridisation signals were quantified on a scanning densitometer (CS-910, Shimadzu Co., Kyoto, Japan).

### Results

*Expression of human recoverin gene in various human lung cancer cell lines*

The expression of recoverin mRNA in ten SCLC cell lines (Figure 1a) and four non-SCLC cell lines (Figure 1b) was tested by RT–PCR. Recoverin mRNA was detected in only one cell line, MN-1112, which was derived from an SCLC of a CAR patient. The additional PCR cycles (up to 40 cycles) did not affect the result, exhibiting no recoverin mRNA signal in the other lung cancer cell lines. The quality of cDNA synthesised in these samples was assured by successful amplification of a $\beta$-actin-specific gene, and the expression level of the $\beta$-actin gene was almost constant in all SCLC cell lines tested.

We also sequenced the entire coding region of recoverin cDNA from MN-1112. A comparison of this sequence with the human retinal recoverin cDNA revealed a 100% identity in the nucleic acid sequence, which contains a myristoilated region at the N-terminus and three calcium binding sites, the so-called EF-hands (data not shown).

The expression of human recoverin mRNA in human lung cancer cell lines was analysed by Northern blotting (Figure 2). A transcript of approximately 1.4 kb was detected in MN-1112. No transcript was detected in three other SCLC cell lines. This transcript was quite similar in size to the published human recoverin mRNA (Murakami *et al*., 1992).

*Detection of recoverin immunoreactivities in the various human SCLC cell lines by Western blots*

The expression of recoverin immunoreactivities in various SCLC cell lines was examined by Western immunoblotting using a polyclonal rabbit anti-S-modulin antibody, which was cross-reacted to the purified bovine recoverin (Figure 3). A prominent single band was detected at a molecular weight region of approximately 23 kDa in only the MN-1112 whole cell lysate. No recoverin-like immunoreactivity was found in any other SCLC cell lines.

### Discussion

The PNSs are frequently associated with SCLC, which possesses many neuronal characteristics (Greco, *et al*., 1981). The neuronal characteristics of SCLC are considered to provide the antigenic stimulus required for production of a cross-reacting immune response to the nervous system targeted (Kornguth, 1989). The expression of some candidates for PNS autoantigen, such as HuD protein for

**Figure 1** Expression of human recoverin and $\beta$-actin mRNAs in SCLC (**a**) and non-SCLC (**b**) cell lines detected by RT–PCR. PCR products were run on a 1.5% agarose gel and visualised with ethidium bromide staining. *Hae*III-digested $\phi$X174DNA was used as a size marker. The molecular size of PCR products was 369 and 504 base pairs (bp). The histological types were SCLC: MN-1112, H-69, N-231, KT-1027, TO-1019, TK-130, MT-428, MN-321, M-319 and LU-134-AM; adenocarcinoma: PC-3 and A-549; squamous cell carcinoma; EBC-1 and VMRC-LCD.

paraneoplastic encephalomyelitis (Sekido *et al.*, 1994) or voltage-gated calcium channels for Lambert–Eaton myasthenic syndrome (Ogura-Okano *et al.*, 1992), has been examined in various SCLC cell lines. However, these autoantigens were expressed in almost all SCLC cell lines tested, both in the cell lines related to PNS and in those not related to PNS. In these cases, to explain rare events of PNS among the common expression of such autoantigens in SCLC, some genetical backgrounds in the cancer host have been proposed which regulate the susceptibility to PNS. On the other hand, in certain PNSs, such a paraneoplastic cerebellar degeneration, the patient-specific expression of neural antigens was demonstrated in tumour tissue from the patient (Furneaux *et al.*, 1990). In this study, we demonstrated that recoverin, the retina-specific protein, is another such antigen for PNSs. Using RT–PCR, we found the gene expression of recoverin, as a conceivable candidate for the CAR autoantigen, exclusively in only one SCLC cell line derived from a patient with CAR (Figure 1). Its immunoreactivity was also found in only this cell line by Western immunoblotting using polyclonal anti-S-modulin antibody (Figure 3). Thirkill *et al.* (1993) previously suggested that recoverin expression could be induced in otherwise quiescent SCLC cell lines. However, the expression of recoverin in an SCLC cell line, MN-1112, was not induced during the establishment and passage of the cell line because, as previously reported, the recoverin expression was immunohistochemically demonstrated in a primary SCLC tumour of the CAR patient from which the cell line was derived (Matsubara *et al.*, 1996).

**Figure 2** Northern blot analysis of human recoverin mRNA in SCLC cell lines. Northern blot filter containing 10 μg of total RNA per lane was hybridised and separated on a 0.4 M formaldehyde–1% agarose gel. (**a**) Hybridised with the $^{32}$P-labelled human recoverin cDNA probe; arrow points to the transcript of about 1.4 kb. (**b**) The same blot hybridised with the human β-actin probe. The size markers of 18S and 28S ribosomal RNAs are indicated.

Previous reports indicated that the presence of autoantibodies to 23 kDa CAR antigen was highly specific for cancer, especially for SCLC. Namely, there have been no reports indicating a correlation between the antibody against 23 kDa peptide and other forms of retinopathy including retinitis pigmentosa, diabetic retinopathy and age-related macular degenerations, in which a variety of antibodies are produced against retinal antigens (Thirkill *et al.*, 1993). Furthermore, a strong antigenicity of recoverin has already been proven by the experiment that an injection of purified recoverin into Lewis rats induced both cellular and humoral immune activation, resulting in an experimental uveoretinitis (Adamus *et al.*, 1994; Gery *et al.*, 1994). Both our and these previous findings strongly suggest that the ectopic expression of recoverin in SCLC cells could sensitise T cells in the cancer host and also stimulate the production of autoantibody from B cells, then induce the degeneration of retina. However, whether a CAR patient possesses a genetically determined susceptibility to induce such an immune response remains unknown. It seems unlikely that the presence of an autoantibody only reflects a non-specific secondary response to retinal injury of other causes.

A spliced or point-mutated form of the responsible antigen expressed in the tumours has been suggested to trigger an autoimmune response in PNS (Dalmau *et al.*, 1992). However, our findings obtained from Northern blotting and sequencing

**Figure 3** Western blot analysis of recoverin in various SCLC cell lines. Whole cell lysates (30 μg) and purified bovine recoverin (2 μg) were separated on SDS/polyacrylamide (10–20% gradient gel) electrophoresis and transferred onto a Clearblot P membrane. The membrane was incubated with a polyclonal rabbit anti-S modulin antibody, followed by goat anti-rabbit IgG coupled with alkaline phosphatase (**b**). Proteins were visualised with Coomassie brilliant blue (**a**). Arrow indicates a band of recoverin. Molecular masses of marker proteins (kilodaltons; kDa) are indicated on the left.

of recoverin in the cells revealed no alternative splicing and no point mutations in the entire coding region (Murakami *et al.*, 1992). This is in line with the above-mentioned animal model, in which the uveoretinitis could be experimentally induced by a purified wild-type form of recoverin.

In conclusion, our findings, together with the previous reports, strongly support the hypothesis that autoimmunity against the ectopic recoverin expressed in SCLC cells cross-reacts with the corresponding antigen located in the retina and induces retinal degeneration in a CAR patient.

**Acknowledgements**
We would like to thank Dr Kawamura for kindly donating the anti-S-modulin antibody, Dr Hitoyasu Futami for donating the H-69 and N-231 SCLC cell lines and Dr Michiaki Tokuda for valuable comments.

### References

ADAMUS G, ORTEGA H, WITKOWSKA D AND POLANS AS. (1994). Recoverin: a potent uveitogen for the induction of photoreceptor degeneration on Lewis rats. *Exp. Eye Res.*, **59**, 447–456.

DALMAU J, FURNEAUX HM, CORDON-CARDO C AND POSNER JB. (1992). The expression of the Hu (paraneoplastic encephalomyelitis/sensory neuropathy) antigen in human normal and tumor tissues. *Am. J. Pathol.*, **141**, 881–886.

FURNEAUX HM, ROSENBLUM MK, DALMAU J, WONG E, WOODRUFF P, GRAUS F AND POSNER JB. (1990). Selective expression of Purkinje-cell antigens in tumor tissue from patients with paraneoplastic cerebellar degeneration. *N. Engl. J. Med.*, **322**, 1844–1851.

FUJITA T, YAMAJI Y, SATO M, MURAO K AND TAKAHARA J. (1994). Gene expression of somatostatin receptor subtypes, SSTR1 and SSTR2, in human lung cancer cell lines. *Life Sci.*, **55**, 1797–1806.

GERY I, CHANAUD NP AND ANGLADE E. (1994). Recoverin is highly uveitogenic in Lewis rats. *Invest. Ophthalmol. Vis. Sci.*, **35**, 3342–3345.

GRECO FA, HAINSWORTH J, SISMANI A, RICHARDSON RL, HANDE KR AND OLDHAM RK. (1981). Hormone production and para-neoplastic syndromes. In *Small Cell Lung Cancer*, Greco FA, Oldham RK and Bunn PA. (eds). pp. 177–224, Grune & Stratton: New York.

KAWAMURA S. (1994). Photoreceptor light-adaptation mediated by S-modulin, a member of a possible regulatory protein family of protein phosphorylation in signal transduction. *Neurosci. Res.*, **20**, 293–298.

KORNGUTH S. (1989). Neuronal proteins and paraneoplastic syndromes. *N. Engl. J. Med.*, **321**, 1607–1608.

MATSUBARA S, SATO M, MIZOBUCHI M, NIIMI M AND TAKAHARA J. (1995). Differential gene expression of growth hormone (GH)-releasing hormone (GRH) and GRH receptor in various rat tissues. *Endocrinology*, **136**, 4147–4150.

MATSUBARA S, YAMAJI Y, FUJITA T, KANAYAMA T, YAMADORI I, SATO M, FUJITA J, SHIOTANI T AND TAKAHARA J. (1996). Cancer-associated retinopathy syndrome: a case of small cell lung cancer expressing recoverin immunoreactivity. *Lung Cancer* (in press).

MURAKAMI A, YAJIMA T AND INANA G. (1992). Isolation of human retinal genes: Recoverin cDNA and gene. *Biochem. Biophys. Res. Commun.*, **187**, 234–244.

OGURA-OKANO M, GRIESMANN GE, WIEBEN ED, SLAYMAKER S, SNUTCH TP AND LENNON VA. (1992). Molecular diversity of neuronal-type calcium channels identified in small cell lung carcinoma. *Mayo Clin. Proc.*, **67**, 1150–1159.

POLANS AS, BUCZYLKO J, CRABB J AND PALCZEWSKI K. (1991). A photoreceptor calcium binding protein is recognized by autoantibodies obtained from patients with cancer-associated retinopathy. *J. Cell Biol.*, **112**, 981–989.

POLANS AS, WITKOWSKA D, HALEY TL, AMUNDSON D, BAIZER L AND ADAMUS G. (1995). Recoverin, a photoreceptor-specific calcium-binding protein, is expressed by the tumor of a patient with cancer-associated retinopathy. *Proc. Natl Acad. Sci. USA*, **92**, 9176–9180.

SATO M AND FROHMAN LA. (1993). Differential effects of central and peripheral administration of growth hormone (GH) and insulin-like growth factor on hypothalamic GH-releasing hormone and somatostatin gene expression in GH-deficient dwarf rats. *Endocrinology*, **133**, 793–799.

SEKIDO Y, BADER SA, CARBONE DP, JOHNSON BE AND MINNA JD. (1994). Molecular analysis of the HuD gene encoding a paraneoplastic encephalomyelitis antigen in human lung cancer cell lines. *Cancer Res.*, **54**, 4988–4992.

THIRKILL CE, FITZGERALD P, SEGOTT RC, ROTH AM, TYLER NK AND KELTNER JL. (1989). Cancer-associated retinopathy (CAR syndrome) with antibodies reacting with retino, optic-nerve and cancer cells. *N. Engl. J. Med.*, **321**, 1589–1594.

THIRKILL CE, TAIT RC, TYLER NK, ROTH AM AND KELTNER JL. (1992). The cancer-associated retinopathy antigen is a recoverin-like protein. *Invest. Ophthalmol. Vis. Sci.*, **33**, 2768–2772.

THIRKILL CE, KELTNER JL, TYLER NK AND ROTH AM. (1993). Antibody reactions with retina and cancer-associated antigens in 10 patients with cancer-associated retinopathy. *Arch. Ophthalmol.*, **111**, 931–937.

YAMAJI Y, MATSUBARA S, YAMADORI I, SATO M, FUJITA T, FUJITA J AND TAKAHARA J. (1996). Characterization of a small cell lung carcinoma cell line from a patient with cancer-associated retinopathy. *Int. J. Cancer*, **65**, 671–676.